# Long noncoding RNA SNHG8 accelerates acute gouty arthritis development by upregulating AP3D1 in mice

**DOI:** 10.1080/21655979.2021.1995579

**Published:** 2021-12-07

**Authors:** Li Fang, Xiangfeng Xu, Yao Lu, Yanying Wu, Jiajia Li

**Affiliations:** Department of Rheumatology and Immunology, Zhoushan Hospital of Zhejiang Province, Zhoushan, Zhejiang, China

**Keywords:** SNHG8, miR-542-3p, AP3D1, acute gouty arthritis

## Abstract

Gout can affect the quality of life of patients due to monosodium urate monohydrate (MSU) crystals. Numerous studies have proposed that long noncoding RNAs (lncRNAs) regulate gout. We aimed to reveal the function of lncRNA small nucleolar RNA host gene 8 (SNHG8) in acute gouty arthritis (GA). A GA mouse model was established by injection of MSU into footpads. The levels of SNHG8, miR-542-3p and adaptor-related protein complex 3 subunit delta 1 (AP3D1) in footpads were detected via polymerase chain reaction analysis. Hematoxylin–eosin staining revealed the paw swelling in mice. Enzyme-linked immunosorbent assay and western blot analysis were applied to determine the concentrations of proinflammatory cytokines. SNHG8 expression was identified to be upregulated after MSU treatment. Ablation of SNHG8 decreased the MSU-induced enhancement of paw swelling and foot thickness. In addition, SNHG8 depletion decreased the protein levels of proinflammatory factors in GA mice. Mechanically, SNHG8 was verified to be a sponge of miR-542-3p, and miR-542-3p targeted AP3D1 3ʹ untranslated region. SNHG8 competitively bound with miR-542-3p to upregulate AP3D1 expression. Finally, results of rescue assays illustrated that AP3D1 upregulation offset the SNHG8-mediated inhibition on paw swelling and protein levels of proinflammatory factors in GA mice. In conclusion, SNHG8 accelerates acute GA development by upregulating AP3D1 in an miR-542-3p-dependent way in mice, providing an effective therapeutic approach to treat acute GA.

## Introduction

Gout is a prevalent disease that is considered as an extremely painful arthritis [1]. The pathogenic process of gout is often accompanied with various comorbidities, including cardiovascular diseases, obesity, diabetes, chronic kidney diseases, and hypertension [2]. Gout is caused by deposition of monosodium urate (MSU) crystals, a crystallized form of uric acid, in joints and periarticular soft tissues due to hyperuricemia [3]. Moreover, mononuclear cells can phagocytose the MSU crystals that were released from pre-formed deposits within the joint, which further induces the productions of proinflammatory factors including tumor necrosis factor alpha (TNF-α), interleukin 1-beta (IL-1β), IL-6, and IL-8 [4,5]. The therapeutic effects of current treatment methods for gout are unfavorable, and it is urgent to identify novel molecular targets to change the status quo.

Long noncoding RNAs (lncRNAs) possess more than 200 nucleotides without capacity for protein coding [6]. The function of lncRNAs is mostly realized through regulating downstream genes at the transcriptional and post-transcriptional levels [7]. LncRNAs are differentially expressed in age-related inflammatory diseases [8,9]. Up to date, multiple lncRNAs are elucidated to make significant contribution to pathogenesis of gout, for example, HOX transcript antisense RNA knockdown alleviates gouty arthritis by downregulation of NLR family pyrin domain containing 3 (NLRP3) [10]. Metastasis associated lung adenocarcinoma transcript 1 suppresses the monosodium urate-induced inflammation via the miR-876-5p/NLRP3 cascade in gouty arthritis [11]. Yu-Feng Qing *et al*. used bioinformatics to analyze lncRNA expression profiles between patients with gouty arthritis and healthy controls and revealed two novel lncRNAs including TCONS_00004393 and ENST00000566457 as candidate diagnostic biomarkers for acute gouty arthritis [12]. Hence, to explore the abnormally expressed lncRNAs can provide a promising therapeutic target for gout.

However, the functions of many lncRNAs in gout have not been elucidated. We performed polymerase chain reaction (PCR) analysis on foot pad of gouty mice and sham mice to determine expression profiles of small nucleolar RNA host gene (SNHG) family members including SNHG1 [13], SNHG4 [14], SNHG5 [15], SNHG7 [16], SNHG8 [17], SNHG12 [18], SNHG14 [19], SNHG15 [20], SNHG16 [21] that were previously reported to regulate inflammatory response. The experimental results revealed that SNHG8 showed the most significant upregulation in gouty mice compared to sham mice. SNHG8 is widely studied as an oncogenic lncRNA through the competitive endogenous RNA (ceRNA) pattern in cancers including gastric cancer [22] and esophageal squamous cell carcinoma [23]. The ceRNA refers to that lncRNAs competitively bind with microRNAs (miRNAs) to antagonize the suppressive effects of miRNAs on their targeted genes [24]. Moreover, SNHG8 attenuates microglial inflammatory response in ischemic stroke by acting as a ceRNA to regulate the miR-425-5p/sirtuin-1 axis [25].

The current study focused on the function and underlying mechanisms of SNHG8 in regulation of acute gouty arthritis (GA) progression. We hypothesized that SNHG8 can regulate the inflammatory response in GA by certain ceRNA network. Our findings can offer a potential new molecular target for the treatment of acute GA.

## Materials and methods

### Cell culture

The human monocytic THP-1 cell line purchased from Cell Bank of the Chinese Academy of Sciences (Shanghai, China) was cultured in RPMI 1640 medium (Life Technologies) added with 10% fetal bovine serum (FBS), sodium pyruvate (1 mM) and β-mercaptoethanol (0.05 mM). Phorbol ester (200 nM) was used to stimulate THP-1 cells for 72 h to induce the differentiation of THP-1 cells into macrophages.

### Cell transfection

Complementary DNA of SNHG8 and adaptor-related protein complex 3 subunit delta 1 (AP3D1) was synthesized and inserted into the pcDNA3.1 vector for overexpression of SNHG8 or AP3D1. The short hairpin RNAs (shRNAs) targeting SNHG8 and AP3D1 (sh-SNHG8 and sh-AP3D1), miR-542-3p mimics and corresponding negative controls (NC) were synthesized by GenePharma (Shanghai, China). Transfection of shRNAs, miRNA mimics or pcDNA3.1 plasmids was performed via the Lipofectamine 2000 (Invitrogen, USA) for 48 h.

### Preparation of MSU crystals

The preparation of MSU crystals was performed under pyrogen-free conditions as previously described [26]. In short, uric acid (1.0 g; Sigma-Aldrich, USA) was dissolved in boiling water (200 mL) with 1 mol/L NaOH (6.0 mL), and the solution pH was adjusted to 7.2 using HCl. Next, the solution was stirred at room temperature for cooling and stored overnight at 4°C. Finally, the precipitate was filtered from the solution and dried under low heat. The crystals were suspended in phosphate-buffered saline (PBS; 50 mg/mL).

### Animals

Forty C57BL/6 mice (sex: male; age: 8–9 weeks; weight: 22–35 g) were commercially obtained from Experimental Animal Center of Shandong University (Jinan, China). Mice were maintained at controlled environment (temperature: 22°C; humidity: 55%) on a 12 h light-dark cycle with access to food and water. Animal experiments in our study were performed strictly based on the National Institutes of Health published Guide for the Care and Use of Laboratory animals and were approved by the Institutional Animal Care and Use Committee of Zhoushan Hospital of Zhejiang Province (Zhejiang, China). After accommodation for 7 days, mice were divided into 5 groups: (i) Sham; (ii) MSU; (iii) MSU+sh-NC; (iv) MSU+sh-SNHG8; (v) MSU+sh-SNHG8+AP3D1. Three weeks before MSU treatment, mice in the iii–v groups received a single injection of 50 μL of lentivirus (element sequence: hU6-MCS-CBh-gcGFP-IRES-puromycin) expressing sh-NC, sh-SNHG8, and AP3D1 (Shanghai Genechem Biological Technology Co., Ltd.) directly into the footpads at the concentration of 1e+6 TU/mL.

### Establishment of a gouty mouse model

Mice were anesthetized by intraperitoneal injection of a mixture of ketamine (150 mg/kg) and xylazine (10 mg/kg). After that, MSU crystals were injected into the right foot pad (1 mg in 40 μL of PBS) of mice. Sham mice were injected with the same volume of sterile saline into the right foot pad. The foot thickness after MSU treatment for 0, 6, 12, and 24 h was detected with an electronic caliper. After MSU treatment for 24 h, mice were euthanized, and the foot pad tissues were collected. Inflammation parameters were measured post MSU crystal injection for 24 h.

### Reverse transcription quantitative polymerase chain reaction (RT-qPCR)

The total RNA of foot pad tissues or THP-1-derived macrophages was extracted by a TRIzol kit (Invitrogen, USA). To synthesize cDNA, total RNA was reverse transcribed using a prime Script RT reagent Kit (Takara, Japan) and miRNAs qPCR Quantitation Kit (Genepharma, Shanghai, China). RT-qPCR was performed in an ABI7500 sequence detection system (Applied Biosystems, USA) using SYBR Green. Glyceraldehyde-3-phosphate dehydrogenase (GAPDH) or U6 acts as an internal control for SNHG8/AP3D1 or miR-542-3p expression, respectively. The relative expression levels of the interested genes were calculated by the 2^−ΔΔCt^ method [27]. The primers for SNHG8, miR-542-3p and AP3D1 were obtained from RiboBio (Guangzhou, China) and shown as follows. SNHG8 (human): forward (F) 5'-ACACATTACGATGGATGATGGA-3', reverse (R) 5'--AAACTTCGCCCATTACCAC-3'-; SNHG8 (mouse): F 5'-ATGTAAAGCCGATACGGTG-3', R 5'-CTACAGTACCTTAACACTTCCTC-3'; miR-542-3p, F 5'-UGUGACAGAUUGAUAACUGAAA-3', R 5'-GTGCAGGGTCCGAGGT-3'; AP3D1 (human), F 5'CATTGCCAAGAATGA-CGAGG-3', R 5'-GTGATCAAGTAGGAGCTGCA-3';AP3D1 (mouse), F 5'-AAGAAGAGCAAGAAGCCCA-3',R 5'-GTCCTCACCCTTCTTGTCC-3';GAPDH (human), F 5'-TCATTTCCTGGTATGACAACGA-3',GTCTTACTCCTTGGAGGCC-3';GAPDH (mouse), F 5'-ACTCTTCCACCTTCGATGC-3',R 5'-CCGTATTCATTGTCATACCAGG-3';U6 (human), F 5'-ATACAGAGAAAGTTAGCACGG-3',R 5'-GGAATGCTTCAAAGAGTTGTG-3'; U6 (mouse), F 5'-CTCGCTTCGGCAGCACA-3', R 5'-ACGCTTCACGAATTTGCGT-3'.

### Western blot analysis

The collected foot pad tissues were treated with 100 μL of radioimmunoprecipitation assay lysis buffer (Beijing Solarbio Science & Technology Co., Ltd., China) and the concentration of protein was determined by use of a bicinchoninic acid kit (Wuhan Boster Biological Technology Ltd., China). The proteins were separated by 10% polyacrylamide gel electrophoresis and transferred onto a polyvinylidene difluoride membrane (Sigma-Aldrich, USA). By blocking with 5% bovine serum albumin, the membranes were incubated with primary antibodies for AP3D1 (ab115234), IL-6 (ab9324), TNF-α (ab1793), IL-1β (ab9722), and GAPDH (ab181602) at 4°C overnight. All above antibodies were purchased from Abcam Company (Hong Kong, China). On the next day, Tris-buffered saline Tween-20 (TBST) was applied for washing the membranes three times followed by incubation with the corresponding goat anti-rabbit secondary antibody (ab6721; Abcam) at room temperature for 1 h. Finally, the blots were developed with an enhanced chemiluminescence system (JingCai Technologies) with the ImageJ software for analyzing the gray value intensity of each blot.

### Luciferase reporter assay

The putative binding sites of miR-542-3p for SNHG8 as well as AP3D1 were predicted by starBase (http://starbase.sysu.edu.cn). MiR-542-3p cDNA was cloned into the pmirGLO Luciferase miRNA Target Expression Vector (Promega) to form the pmirGLO-miR-542-3p-WT reporter vector. The mutant miR-542-3p was specifically synthesized and inserted into the pmirGLO vector to construct the pmirGLO-miR-542-3p-Mut reporter vector. sh-NC and sh-SNHG8 were transfected with pmirGLO-miR-542-3p-WT or pmirGLO-miR-542-3p-Mut into THP-1-derived macrophages. For validating the interaction of miR-542-3p and AP3D1 3ʹUTR, the wild-type and mutant AP3D1 3ʹ-UTR fragments were cloned into the pmirGLO vector to form pmirGLO-AP3D1-WT and pmirGLO-AP3D1-Mut, respectively. The miR-542-3p mimics, NC mimics, or miR-542-3p mimics + SNHG8 was cotransfected with pmirGLO-AP3D1-WT and pmirGLO-AP3D1-Mut into THP-1-derived macrophages using Lipofectamine 3000 (Invitrogen). After transfection for 48 h, the luciferase assay was performed with the employment of the Luciferase Reporter Assay System (Promega). Relative firefly luciferase activity was normalized to Renilla luciferase activity.

### Nuclear-cytoplasmic fractionation

Isolation of cytoplasmic and nuclear SNHG8 was performed by use of a PARIS™ Kit (Invitrogen, USA). In brief, after lysing with cell fractionation buffer, cells were centrifuged for separating the nuclear and cytoplasmic cell fractions. The supernatant was collected followed by transferring to a fresh RNase-free tube. Cell fractionation buffer was further used for washing the left lysate. To lyse the nuclei, cell disruption buffer was supplemented. Afterward, the lysate and the supernatant were mixed with a 2× lysis/binding solution and ethanol. Thereafter, the mixture was drawn through a filter cartridge. Finally, elution solution was applied for eluting the cytoplasmic and nuclear SNHG8 followed by RT-qPCR analysis.

### RNA immunoprecipitation (RIP) assay

A Magna RNA-binding protein immunoprecipitation kit (Millipore, USA) was used for RIP assay. Briefly, cell lysate was first cultured with RIPA buffer together with magnetic beads conjugated with human Ago2 antibody or IgG antibody. The samples were incubated with Proteinase K and immunoprecipitated RNA was separated. The RNA concentration was detected via a spectrophotometer (Thermo Scientific, USA) and the RNA quality was determined with a bio-analyzer (Agilent, USA). Finally, the extracted purified RNAs were analyzed through RT-qPCR.

### Enzyme-linked immunosorbent assay (ELISA)

IL-6, TNF-α and IL-1β concentrations in foot pad tissues of mice were measured via ELISA (Dakewei Biotech, China). Tissue homogenate samples were seeded in a 96-well ELISA microplate with captured antibody. The captured antibody-bound cytokines were detected by use of biotin-conjugated anti-IL-6, anti-TNF-α and anti-IL-1β antibodies and horseradish peroxidase (HRP)-conjugated avidin. A microplate reader (Bio-Rad, China) was used to read the reaction plates at 450 nm within 30 min. IL-6, TNF-α and IL-1β concentrations were evaluated in accordance with the manufacturer’s instructions.

### Hematoxylin-eosin (H&E) staining

To evaluate the histological analysis, sagittal sections of the footpads were fixed in 10% formalin for 24 h. The tissues were embedded with paraffin and sectioned after decalcification. Finally, the tissues were subjected to H&E staining [28].

### Statistical analysis

SPSS 23.0 (SPSS, USA) was employed to analyze all the experimental statistics. Data were exhibited as the mean ± SD derived from three independent experiments. Student’s t-test (for 2 groups) or one-way ANOVA (for more than 2 groups) was utilized. *P* values less than 0.05 were considered statistically significant.

## Results

### SNHG8 expression in foot pad tissues is increased by MSU

**Figure 1.**
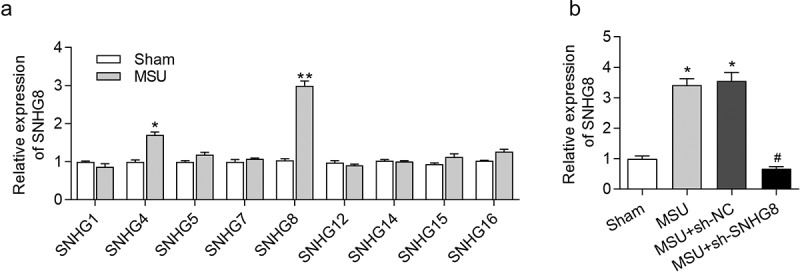
**SNHG8 expression in foot pad is increased by MSU**. (a) RT-qPCR disclosed that MSU treatment significantly enhanced the expression of SNHG4, SNHG8 in foot pad tissues of mice. (b) RT-qPCR manifested that SNHG8 expression in foot pad tissues of GA mice was silenced by injection of lentivirus expressing sh-SNHG8. *p < 0.05, **p < 0.01 *vs*. sham group, ^#^*p* < 0.05 *vs*. MSU+ sh-NC group. There were eight mice in each group

We constructed a GA mouse model by MSU treatment and detetced SNHG8 in foot pad tissues. From the results of RT-qPCR analysis, MSU treatment caused the most significant upregulation of SNHG8 in foot pad tissues (Figure 1a), suggesting that SNHG8 is involved in the development of GA. Afterward, SNHG8 expression in footpad was successfully silenced by injection of lentivirus expressing sh-SNHG8 in MSU-stimulated GA mice (Figure 1b).

### Silencing of SNHG8 suppresses paw swelling and inflammation

**Figure 2.**
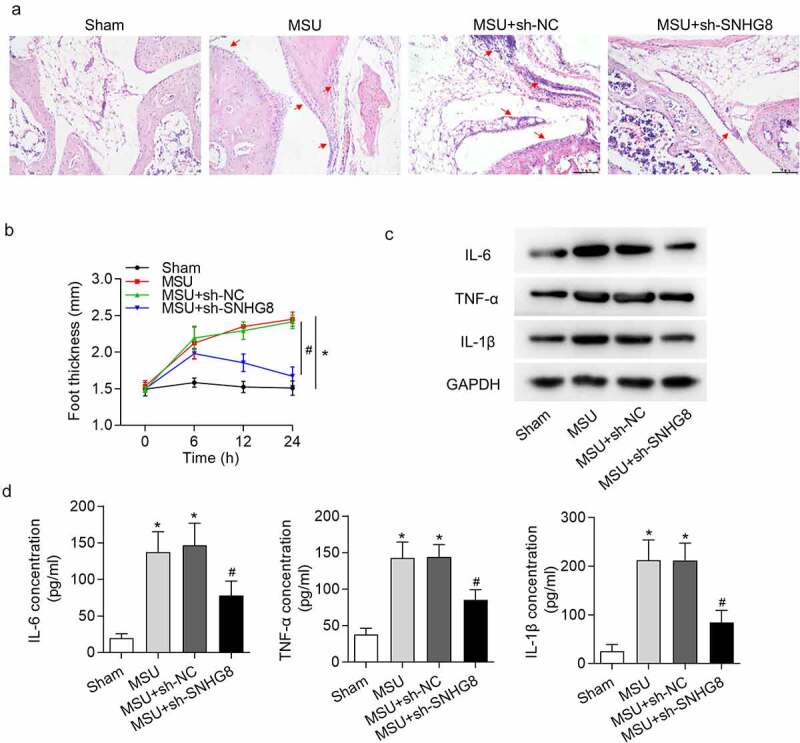
**Silencing of SNHG8 suppresses paw swelling and inflammation**. (a) H&E staining disclosed that MSU-induced paw swelling was rescued by SHNG8 depletion. Red arrows indicate the inflammatory cells. (b) The foot thickness changes in mice after MSU stimulation for 0, 6, 12, 24 h were exhibited. (c) Protein levels of IL-6, TNF-α and IL-1β in foot pad tissues were enhanced by MSU stimulation and decreased by SNHG8 deficiency, as suggested by western blot analysis. (d) ELSIA results revealed that MSU significantly increased the concentrations of IL-6, TNF-α and IL-1β and silenced SNHG8 decreased their concentrations. *p < 0.05 *vs*. sham group. ^#^*p* < 0.05 *vs*. MSU + sh-NC group. There were eight mice in each group

Furthermore, the effect of SNHG8 on MSU-induced GA mice was assessed. GA can result in edema in mice paw, and the extent of edema was detected. H&E staining results disclosed that MSU significantly increased paw swelling while this was rescued by SHNG8 depletion (Figure 2a). The foot thickness changes at 0, 6, 12, 24 h were exhibited in Figure 2b. Moreover, inflammation is an index of GA, and thus proinflammatory cytokines IL-1β, TNF-α and IL-6 were evaluated following injection of MSU. According to the data from western blot analysis, MSU significantly increased IL-6, TNF-α and IL-1β protein levels, while after injection of SNHG8-silenced lentiviral vector, the concentrations of IL-6, TNF-α and IL-1β were declined (Figure 2c). The results from ELISA were in accordance with western blot analysis, revealing that IL-6, TNF-α and IL-1β protein levels were enhanced by MSU but decreased by sh-SNHG8 (Figure 2d).

### SNHG8 served as a sponge of miR-524-3p

**Figure 3.**
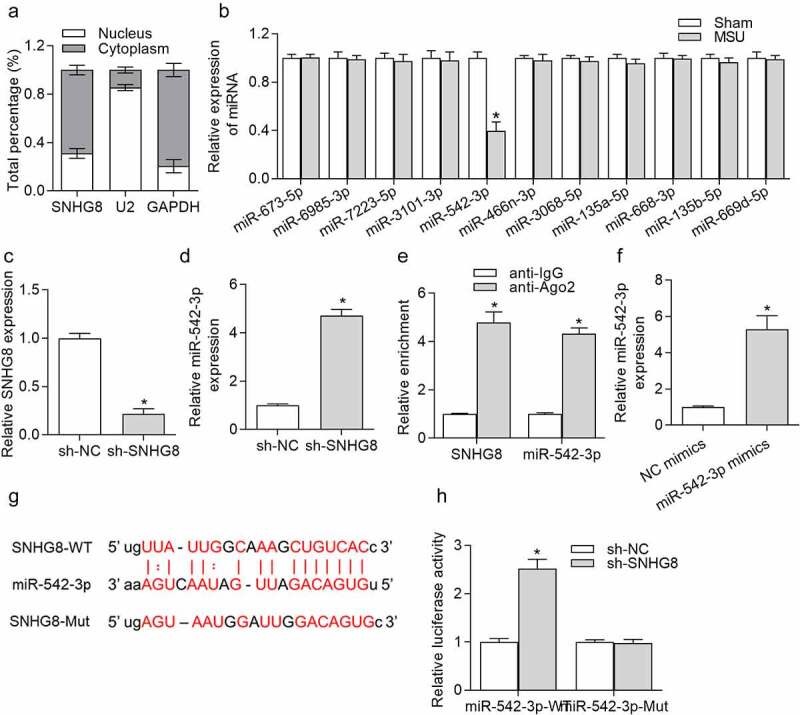
**SNHG8 served as a sponge of miR-524-3p**. (a) Subcellular fraction assay showed that SNHG8 was mostly distributed in the cytoplasm of THP-1-derived macrophages. (b) Expression of miRNAs in foot pad tissues of mice after MSU treatment was detected by RT-qPCR. *p < 0.05 *vs*. sham group. There were eight mice in each group. (c) SNHG8 knockdown efficiency in THP-1-derived macrophages was verified by RT-qPCR. (d) RT-qPCR manifested that miR-542-3p expression was significantly enhanced due to SNHG8 silence in THP-1-derived macrophages. *p < 0.05 *vs*. sh-NC group. (e) RIP assay was conducted to reveal that both SNHG8 and miR-542-3p were enriched in anti-Ago2 group. *p < 0.05 *vs*. anti-IgG group. (f) RT-qPCR manifested that miR-542-3p was overexpressed by transfection of miR-542-3p mimics in THP-1-derived macrophages. *p < 0.05 *vs*. NC mimics group. (g) Bioinformatics analysis predicted the binding sites for SNHG8 and miR-542-3p. (h) Luciferase reporter assay was utilized to further identified the binding possibility between SNHG8 and miR-542-3p. *p < 0.05 *vs*. NC mimics group. Each biological sample was run in triplicate and experiments were independently repeated three times for the *in vitro* studies

Given that lncRNAs can serve as ceRNAs to competitively bind with miRNAs to regulate specific gene expression and thereby influence the progression of diseases [29], we speculated that SNHG8 serves as a ceRNA in GA, and its downstream miRNAs were explored. Subcellular fraction assay was conducted in THP-1-derived macrophages. We observed that SNHG8 was mostly (73%) distributed in the cytoplasm (Figure 3a), indicating that SNHG8 had the potential to act as a ceRNA to modulate miRNAs. The starBase online tool [30] was applied for identifying candidate miRNAs that were able to be sponged by SNHG8, showing that miR-542-3p expression was decreased in foot pad tissues after MSU treatment for 24 h, while other miRNAs exhibited no significant changes after MSU (Figure 3b), implying that SNHG8 might regulate GA progression by interaction with miR-542-3p. Figure 3c reveals that transfection of sh-SNHG8 for 48 h effectively reduced SNHG8 expression by 78.5% in THP-1-derived macrophages. RT-qPCR analysis manifested that miR-542-3p expression was significantly enhanced due to SNHG8 silence (Figure 3d). Further, to validate whether SNHG8 can sponge miR-542-3p in THP-1-derived macrophages, RIP assay was conducted. Both SNHG8 and miR-542-3p were enriched in anti-Ago2 group (Figure 3e), suggesting that SNHG8 and miR-542-3p coexist in the same RNA-induced silencing complex (RISC). Next, miR-542-3p was effectively overexpressed (by 4.5 folds) by transfection of miR-542-3p mimics in THP-1-derived macrophages, resulting in a markedly elevation of miR-542-3p expression (Figure 3f). starBase predicted the binding sites for SNHG8 and miR-542-3p, and we mutated the binding sites for luciferase reporter assay (Figure 3g). Based on the results of luciferase reporter assay, we found that silencing of SNHG8 had no significant effects on the luciferase activity of miR-542-3p-Mut, but remarkably decreased the luciferase activity of miR-542-3p-WT (Figure 3h), confirming that SNHG8 could sponge miR-542-3p.

### AP3D1 was a direct target of miR-542-3p

**Figure 4.**
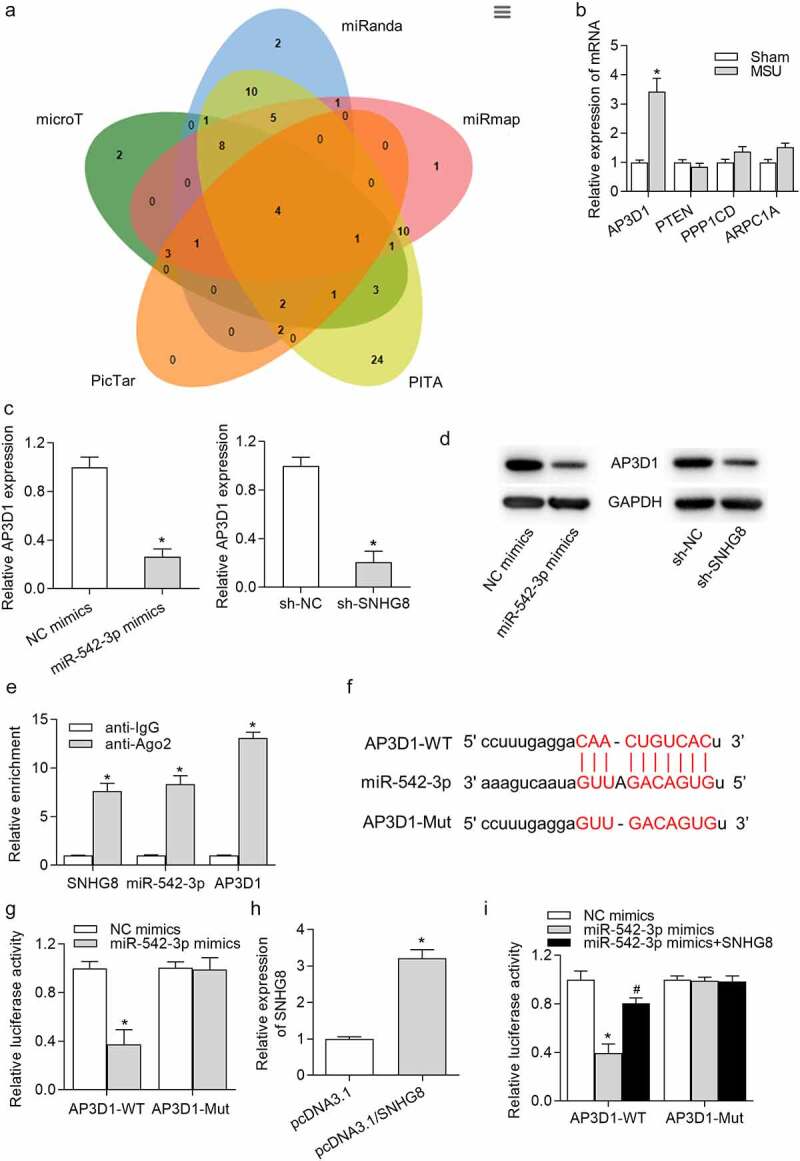
**AP3D1 was a direct target of miR-542-3p**. (a) Venn diagram showed that there were four potential targets of miR-542-3p, including AP3D1, PTEN, PPP1CD and ARPC1A, according to data from PicTar, PITA, miRmap, miRanda and microT databases. (b) RT-qPCR showed that 24 h of MSU treatment increased AP3D1 expression and had no effects on the expression of PTEN, PPP1CD and ARPC1A in foot pad tissues of mice. *p < 0.05 *vs*. sham group. There were eight mice in each group. (c) RT-qPCR demonstrated that AP3D1 expression was declined by miR-542-3p upregulation or SNHG8 ablation in THP-1-derived macrophages. *p < 0.05 *vs*. NC mimics or sh-NC group. (d) Western blot analysis indicated that the protein level of AP3D1 was decreased by overexpression of miR-542-3p or downregulation of SNHG8. (e) The coexistence of SNHG8, miR-542-3p and AP3D1 in RISC was confirmed by RIP assay. *p < 0.05 *vs*. anti-IgG group. (f) The potential binding site for miR-542-3p and AP3D1 3ʹUTR was shown based on the starBase online tool. (g) The binding capacity of miR-542-3p and AP3D1 3ʹUTR was confirmed by luciferase reporter assay. *p < 0.05 *vs*. NC mimics group. (h) RT-qPCR manifested that SNHG8 expression was overexpressed by pcDNA3.1/SNHG8. *p < 0.05 *vs*. pcDNA3.1 group. (i) Luciferase reporter assay was conducted to detect the luciferase activity of AP3D1-WT and AP3D1-MUT in cells transfected with NC mimics, miR-542-3p mimics, or cotransfected with miR-542-3p mimics+SNHG8. *p < 0.05 *vs*. NC mimics group, *#p* < 0.05 *vs*. miR-542-3p mimics group. Each biological sample was run in triplicate and experiments were independently repeated three times for the *in vitro* studies

Next, the targets downstream miR-542-3p were investigated. PicTar, PITA, miRmap, miRanda and microT databases were employed to identify the specific genes that can be targeted by miR-542-3p. Venn diagram shown that there are four potential targets (AP3D1, PTEN, PPP1CD, and ARPC1A) of miR-542-3p (Figure 4a). However, RT-qPCR revealed that 24 h of MSU treatment increased AP3D1 expression and had no significant effects on mRNA levels of PTEN, PPP1CD and ARPC1A (Figure 4b). RT-qPCR analysis demonstrated that AP3D1 expression was declined by miR-542-3p upregulation or SNHG8 ablation (Figure 4c). Consistently, the protein level of AP3D1 was decreased by miR-542-3p overexpression or downregulation of SNHG8 (Figure 4d). Next, we evaluated whether miR-542-3p binds to AP3D1 by RIP and luciferase reporter assays. RIP assay results disclosed that SNHG8, miR-542-3p and AP3D1 were abundantly enriched in the Ago2 pellet (Figure 4e). Moreover, the predicted binding site for miR-542-3p and AP3D1 3ʹUTR based on starBase was presented in Figure 4f, and we mutated the binding sites. Afterward, the luciferase gene reporter vector pmiRGLO-AP3D1-WT and pmiRGLO-AP3D1-Mut were constructed and co-transfected with NC mimics or miR-542-3p mimics into THP-1-derived macrophages. We observed that there was a significant increase of the luciferase activity of pmiRGLO-AP3D1-WT instead of pmiRGLO-AP3D1-Mut (Figure 4g), indicating the interaction of miR-542-3p and AP3D1 3ʹUTR. Further, we successfully overexpressed SNHG8 expression by transfection of pcDNA3.1/SNHG8 into THP-1 derived macrophages (Figure 4h). Luciferase reporter assay showed that SNHG8 upregulation reversed the miR-542-3p-mediated decline of the luciferase activity of pmiRGLO-AP3D1-WT and had no influence on the luciferase activity of pmiRGLO-AP3D1-Mut (Figure 4i), implying that SNHG8 bound with miR-542-3p to upregulate AP3D1.

### SNHG8 contributed to GA progression by upregulating AP3D1

**Figure 5.**
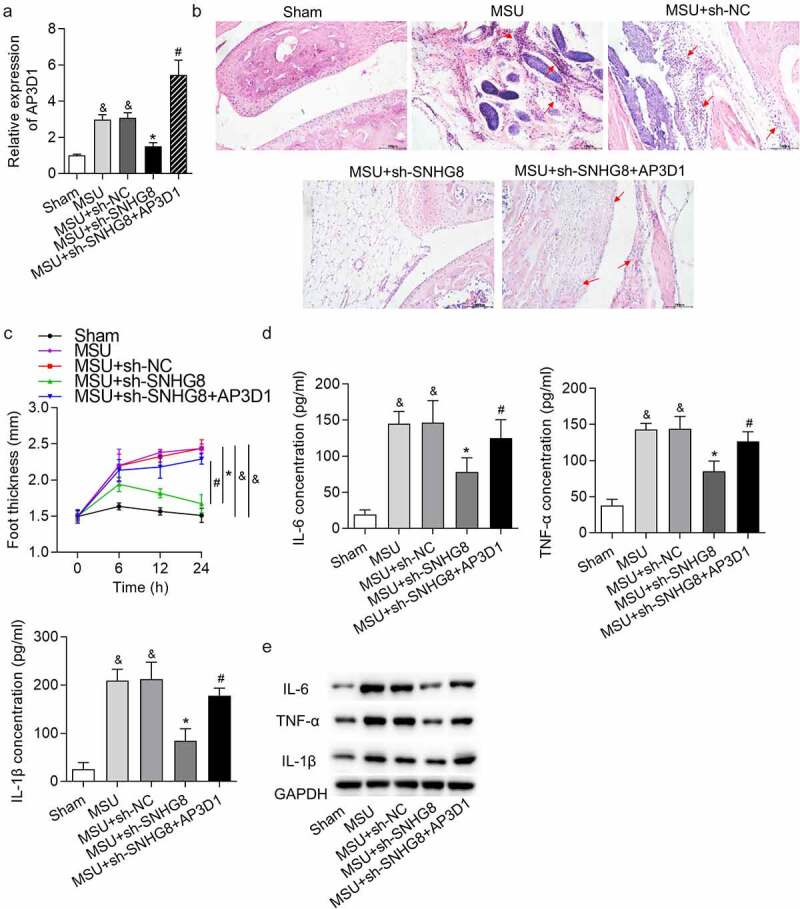
**SNHG8 contributed to GA progression by upregulating AP3D1**. (a) RT-qPCR showed that AP3D1 was upregulated in foot pad tissues of an MSU-induced GA mice model. (b) H&E staining showed that AP3D1 upregulation rescued the inhibitory impact of SNHG8 deficiency on mice paw swelling. Red arrows indicate the inflammatory cells. (c) The changes of foot thickness were displayed in the curve graphs. (d-e) ELISA and western blot analysis showed that upregulation of AP3D1 countervailed the SNHG8 depletion-triggered inhibition on IL-6, TNF-α and IL-1β levels in foot pad tissues. ^&^*p* < 0.05 *vs*. sham group, *p < 0.05 *vs*. MSU + sh-NC group, ^#^*p* < 0.05 *vs*. MSU + sh-SNHG8 group. There were eight mice in each group

Subsequently, rescue experiments were performed to confirm whether SNHG8 promotes GA progression by upregulating AP3D1. AP3D1 expression was upregulated in MSU-induced GA mice, and was decreased by SNHG8 silence, while injection of lentivirus expressing AP3D1 increased AP3D1 expression (Figure 5a). H&E staining showed that AP3D1 upregulation rescued the inhibitory impact of SNHG8 deficiency on mice paw swelling (Figure 5b) and the changes of foot thickness were displayed in Figure 5c. Moreover, upregulation of AP3D1 countervailed the SNHG8 depletion-triggered inhibition on IL-6, TNF-α and IL-1β protein levels, as supported by ELISA and western blotting results (Figure 5(d–e)). In general, SNHG8 contributed to GA progression by upregulating AP3D1.

## Discussion

Gout is an inflammatory arthritis induced by the deposition of MSU crystals in and around the joints [31]. In the present study, SNHG8 expression was upregulated in the footpads of an MSU induced mouse model of GA. Additionally, silencing of SNHG8 significantly reduced the MSU-induced increased foot thickness in mice. It is reported that gout is closely related to inflammatory response and regulates the activation of typical inflammatory pathways such as toll-like receptor (TLR) 4-NFkappaB-IL-1β signaling [32], and NLRP3-IL-1β signaling pathway [33]. Thus, to explore the status of inflammatory factors in acute gouty arthritis is necessary. In this study, SNHG8 depletion significantly decreased the protein levels of proinflammatory factors including IL-1β, TNF-α and IL-6 in footpads of gouty mice. In summary, the data above indicated that silencing of SNHG8 alleviated the development of GA by suppression on inflammatory response. Oligonucleotide therapies have promising potentials in arthritis treatment [34]. Our study indicated that using oligonucleotides to knock down SNHG8 may have positive effects on GA treatment.

Among all the functional mechanisms of lncRNAs, the ceRNA hypothesis at the post-transcriptional level is widely accepted to explain how lncRNAs work out in many diseases [35]. SNHG8 has been confirmed to act as a ceRNA in cancers including esophageal squamous cell carcinoma [23] and gastric cancer [36]. Therefore, we hypothesized that SNHG8 is involved in the ceRNA network in GA. Macrophages are essential for MSU-induced inflammation in GA [37]. We found that SNHG8 majorly exist in the cytoplasm of THP-1-derived macrophages, indicating the post-transcriptionally regulatory effect of SNHG8 on macrophages and GA. We then predicted the miRNAs which bind to SNHG8 through bioinformatic analysis. Subsequently, expression of candidate miRNAs in footpads of mice after MSU treatment was detected, and the results disclosed that miR-542-3p expression was decreased in footpads of GA mice. Previously, miR-542-3p was proposed to regulate the inflammatory response in cerebral injury [38], coronary heart disease [39], and respiratory viral infections [40]. In the present study, we found that SNHG8 binds with miR-542-3p and negatively regulates its expression in THP-1-derived macrophages. However, the exact role and function of miR-542-3p in GA were unclear and further studies will be conducted to investigate this issue.

Furthermore, bioinformatic analysis revealed that AP3D1 is a target downstream miR-542-3p. AP-3 is a heterotetrametric adaptor protein complex reversibly combining with early endosomal membranes, recognizes and packages transmembrane proteins into clathrin-coated transport vesicles for delivery to the lysosomes or lysosome-related organelles [41]. AP3D1 mutation is associated with immunodeficiency and seizures and defines a new type of Hermansky-Pudlak syndrome [42]. We found that, in MSU-stimulated GA mice, AP3D1 expression was significantly increased. Moreover, the mRNA and protein levels of AP3D1 were negatively regulated by miR-542-3p and positively regulated by SNHG8. Furthermore, we found that AP3D1 3ʹUTR was targeted and degraded by miR-542-3p in THP-1-derived macrophages. It can be included that SNHG8 bound with miR-542-3p to suppress the degradative effects of miR-542-3p on AP3D1. Finally, rescue assays revealed that AP3D1 upregulation inversely changed the SNHG8 ablation-mediated inhibition on foot thickness and protein levels of IL-6, TNF-α and IL-1β.

## Conclusion

This study revealed that SNHG8 promotes acute GA development in mice by upregulating AP3D1 through acting as a ceRNA to regulate miR-542-3p, suggesting SNHG8 as a potential candidate in the prevention and treatment of GA.
